# FAMILY CAREGIVER SCREENING IN PRIMARY CARE: CLINICIAN AND CAREGIVER PERSPECTIVES

**DOI:** 10.1093/geroni/igz038.750

**Published:** 2019-11-08

**Authors:** Catherine Riffin

**Affiliations:** Weill Cornell Medicine, New York, New York, United States

## Abstract

Despite broad appreciation of family caregivers’ relevance to older adults’ health care, few primary care-based interventions have incorporated mechanisms to facilitate systematic caregiver identification, screening, and support. Actionable knowledge regarding how such interventions may be incorporated into clinical practice is remarkably limited. This study used in-depth interviews to elucidate clinicians’ (N=25) and caregivers’ (N=20) perspectives on and suggestions for integrating caregiver screening into primary care practice. Transcripts were analyzed using qualitative content analysis. Participants emphasized the importance of tailoring the caregiver screening intervention to local circumstances and to patient and caregiver preferences. They advocated for an action-oriented approach that would link identified risks with a concrete plan for follow-up (e.g., referral to training) and outcomes relevant to the patient’s care plan. Overall, participants advised that integrating the intervention into practice would require the support of multidisciplinary practice staff, stronger connections between medical and community-based services, and appropriate reimbursement for clinicians.

